# Breastfeeding Duration: A Survival Analysis—Data from a Regional Immunization Survey

**DOI:** 10.1155/2014/529790

**Published:** 2014-06-04

**Authors:** E. Robert, Y. Coppieters, B. Swennen, M. Dramaix

**Affiliations:** ^1^Research Center of Health Policy and Systems-International Health, School of Public Health, Université Libre de Bruxelles, Route de Lennik 808, 1070 Brussels, Belgium; ^2^Research Center of Epidemiology, Biostatistics and Clinical Research, School of Public Health, Université Libre de Bruxelles, Route de Lennik 808, 1070 Brussels, Belgium

## Abstract

*Objective*. To report the duration of and factors associated with exclusive and any breastfeeding among the French-speaking community of Belgium (Wallonia). *Material and Methods*. A two-stage cluster sample was drawn from the population of children aged 18–24 months living in the area in 2012. Anamnestic data on breastfeeding and sociodemographic information were collected from 525 mothers. Cox's proportional hazards model was used to identify factors associated with discontinuing breastfeeding. *Results and Discussion*. Only 35.1% of the women were satisfied with their duration of any breastfeeding. At 3 months, 54.1% of the infants were breastfed, of which 40.6% exclusively, with these percentages falling to 29.1% and 12.6% at 6 months. Exclusive and any breastfeeding durations were independently positively associated (*P* < 0.05) with foreign-born mothers, awareness of WHO recommendations, and maternity leave >3 months. Exclusive BF duration was associated with higher parental income and the prenatal decision to breastfeed. The duration of any breastfeeding was associated with the mothers' age of ≥30 years and whether they were exclusively breastfeeding at discharge from the maternity unit. *Conclusions*. Programs promoting and supporting BF should concentrate on training prenatal health-care professionals. Prenatal professional advice may promote adherence to WHO BF guidelines. The benefits of exclusive BF should be emphasized. Pregnant women should be discouraged from introducing supplementary feeding in the maternity ward.

## 1. Introduction

Worldwide, the recommendation since 2003 has been for mothers to exclusively breastfeed their babies for the first 6 months and to continue to breastfeed up to 2 years of life [1]. The obvious benefits of breastfeeding (BF) for both child and mother are fully described [2].

Great differences exist in BF prevalence and duration both within and between European countries [3]. On the basis of data prior to 2000, Cattaneo et al. gave, for Belgium, BF rates that were among the lowest in Europe: 30–37% at 3-4 months, 10% at 6 months, and 4% at 12 months [4]. In 2001, Yngve and Sjöström wrote that Belgium, France, the Netherlands, and Italy seemed to be the EU countries with the greatest challenges ahead. Indeed, data collection, promotion, and policy are particularly limited in these countries [3]. The Office of Economic Cooperation and Development (OECD) shows that Belgium, France, and Ireland had the worst breastfeeding-at-birth rates in 2005 [5].

The findings of various literature reviews show that BF duration depends on a large number of factors. Older mothers, multiparity, and a higher social class are associated with longer BF durations [6–8]. Full-term pregnancy and vaginal births positively influence BF duration. Some psychosocial factors (knowledge, family support, etc.) are also significant as regards BF duration [9, 10]. Maternity-leave duration also has an impact on the duration of BF [6, 11, 12]. Maternity-leave systems differ across European countries with regard to duration and payments [5]. Maternity leave in Belgium is 15 weeks' not full pay and is one of the shortest in Europe. Belgium, like various others countries, offers the possibility of prolonging the parental leave period.

The Baby-Friendly Hospital Initiative (BFHI) can also play a role in improving BF prevalence and duration [13]. In 2010, 22 maternity units (including 6 in Wallonia) out of the country's 177 units were BFHI accredited. Belgium did not have any BFHI-accredited maternity units in 2003.

The two existing data sources in Wallonia only document breastfeeding at birth. The first source is the maternal and child health (MCH) program, which collects data from the maternity ward. However, not all hospitals allow visits from MCH medical social workers. Therefore, the data collected by this program is not exhaustive. The second source of information comes from the BFHI-accredited hospitals, which collect data on exclusive maternal breastfeeding. This data is incomplete and biased as it is from accredited maternity units. The BFHI protocol and MCH questionnaire contain no socioeconomic data on parents. The BFHI data is not published.

In Belgium, at present, there are still no regular standardized surveys on BF, and no specific studies have been conducted on the duration of BF and its associated factors. It was therefore decided to introduce a breastfeeding module, based on World Health Organization (WHO) definitions, in the vaccination coverage survey, the only survey that is representative of the entire French-speaking community of Belgium and is targeted at young children.

Over the years, BF rates in Wallonia have risen, but they remain low compared with other European countries. Very few mothers achieve the BF duration they would like to achieve. Given these issues, the risk factors associated with weaning need to be determined so as to develop appropriate solutions and possibly strengthen existing programs in order to achieve longer BF durations. We will therefore examine to what extent BF duration in Wallonia is determined by the various characteristics frequently cited in the literature.

## 2. Material and Methods

### 2.1. Population and Sampling

An expanded program on immunization (EPI), two-stage cluster sampling study was conducted between May and July 2012. The list of municipalities for each province was obtained from the Belgian Directorate General for Statistics and Economic Information (DGSE). In the first stage, 55 clusters were selected with probability proportional to size, which allowed larger municipalities to be drawn more than once. A list of children born between May 31 and November 30, 2010, was obtained from each selected community. In the second stage, 12 children in each cluster were randomly selected from the municipality list. Each family selected received a brief information letter announcing that an interviewer would visit them to conduct a survey on “infancy.” Families were substituted with a replacement if they could not be contacted after three attempted visits. All of the children were aged between 18 and 24 months at the time of the survey. Each questionnaire was thoroughly checked by the person responsible for the survey. For each outlier, if necessary, a phone call to the parents was made to verify the information and correct it.

The sample was representative of the French-speaking community of Belgium. Indeed, the sociodemographic data was comparable to the database of the Perinatal Epidemiology Centre (CEPIP) which records and analyzes all statistical reports on births in the region [14]. The region covers the French-speaking area of the country and represents 35% of the Belgian population. The database was registered by the Commission for the Protection of Privacy in Belgium.

### 2.2. Variables Used

A 16-question breastfeeding module was introduced in the survey. Data for BF is retrospective. The questions on BF can be grouped into four categories: (i) the type of BF at birth and at discharge from the maternity ward; (ii) the age of the child when any and exclusive BF was stopped; (iii) the main reasons for stopping any and exclusive BF (open questions); (iv) BF predictors (BFHI, mode of delivery, father's attitude, etc.).

All of the independent variables were dichotomized apart from the parents' level of education and the “age of infant when mother returned to work,” which was broken down into three categories. For this predictor, “≤3 months” corresponds to students and self-employed or employed mothers who settled for the legal maternity-leave period, “4+ months” corresponds to the mothers who took extended parental leave, and the third category includes mothers who were unemployed and consequently did not return to work.

“Exclusive breastfeeding” is defined as the intake of breast milk (directly expressed or from a wet nurse) without any additional liquids or solid/semisolid foods; intakes of oral rehydration solution (ORS), vitamins, minerals, or medications in the form of drops or syrups are allowed [15].

### 2.3. Statistical Analysis

Only mothers who breastfed at birth were included in the analyses. The duration of any and exclusive BF was based on the month during which BF was stopped. The factors associated with any and exclusive BF durations were studied. The median durations and 95% CI of any and exclusive BF were derived using Kaplan-Meier survival curves. The Log-Rank and Breslow tests were used to assess the equality of the survival curves. We performed Cox proportional hazards (PH) models. The covariates included in the multivariable Cox model were selected by a backward stepwise procedure. All variables associated with the duration of BF with* P* values ≤ 0.10 were included in the final model. PH assumption was assessed checking the parallelism of the curves Ln(−Ln(*S*(*t*))), with* S*(*t*) being the survival curve derived from the Cox model. Potential interactions were tested. They were nonsignificant. Epi-Info (6.04d.fr) and SPSS-IBM Statistics 22 were used for encoding and all statistical analyses.

## 3. Results

A total of 525 respondents completed the module on breastfeeding. 480 mothers (91.5%), 37 (7.0%) fathers, and 8 (1.5%) grandmothers responded to the survey. A little under a quarter of the infants had been born in a BFHI-accredited maternity unit. More than 92% of the children had been born at full term, and 78% were born vaginally. Nearly 43% of the children were ranked 1 (Table 1). The majority of the mothers (60.1%) were aged 30 years or older at the time of the survey. Only 21.0% of the mothers had taken just the legal maternity leave of 15 weeks and nearly half (45%) had taken breastfeeding leave or parental leave. The majority of the mothers (71.8%) were of Belgian origin and 65% had made the decision to breastfeed before pregnancy. More than a quarter of the mothers knew the WHO recommendation of 6 months' exclusive breastfeeding (Table 2). Among the mothers who had started BF, only 35.1% were satisfied with the duration of their breastfeeding (not included in table). Nearly one in five fathers was not in favor of breastfeeding (Table 3).

### 3.1. Prevalence of Breastfeeding in the Maternity Unit at 3, 6, and 12 Months

**Figure 1:**
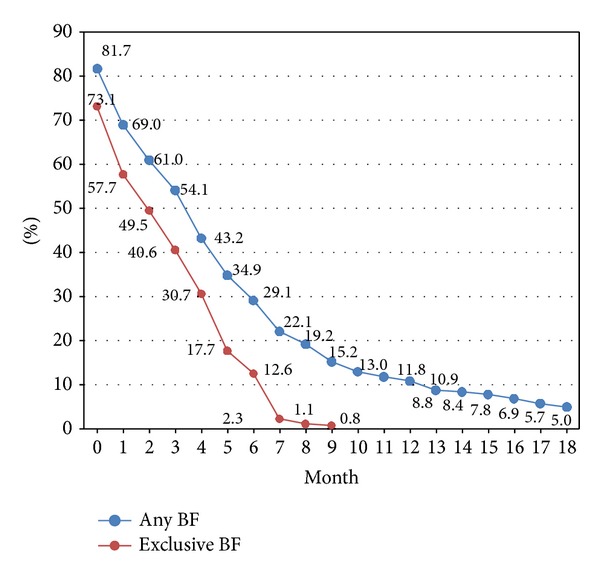
Rates of any and exclusive breastfeeding (%) during the infants' first 18 months of life (*n* = 525).

The median duration of any BF was 4.0 months (min-max) (0.03–21.4) and of exclusive BF 3.0 months (0.03–11.0). The proportion of women breastfeeding at birth was 81.7% (95% CI) (78.4–85.0) with 73.1% (69.4–76.9) exclusively breastfeeding. 18.3% of the women did not breastfeed at all at birth. At discharge from the maternity unit, 76.4% (72.2–79.6) of the women were breastfeeding with 66.4% (62.2–70.3) of infants being exclusively breastfed. At 3 months of age, more than one-half (54.1%) (49.8–58.4) of the infants were receiving some breast milk and 40.6% (36.4–44.8) were being exclusively breastfed. At 6 months of age, 29.1% (25.3–33.0) of the infants were receiving some breast milk and 12.6% (9.7–15.4) were being exclusively breastfed. And at 12 months, the prevalence of BF was 10.9% (8.2–13.5) (Figure 1).

### 3.2. Factors Associated with the Duration of Any and Exclusive Breastfeeding

**Figure 2:**
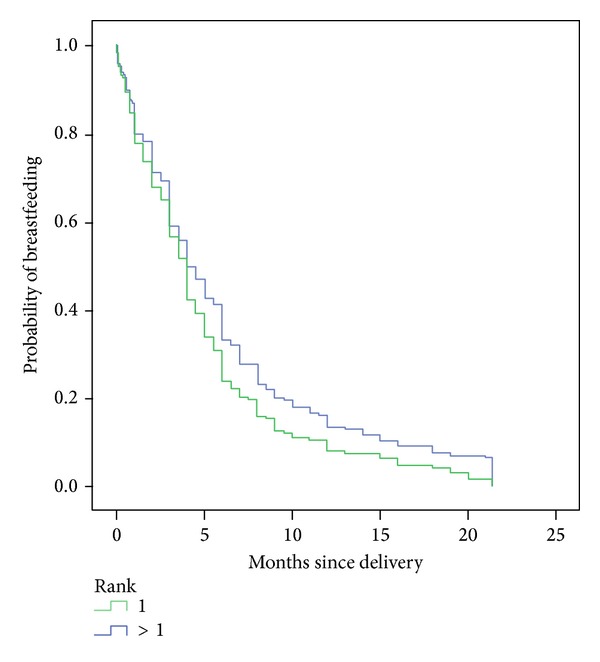
Probability of any breastfeeding depending on duration (months) by children's parity.

Children who were exclusively breastfed at birth were breastfed one and a half months longer than children for whom breast-milk substitutes had been introduced. Children born at full term were breastfed slightly longer (0.5 months) than those born prematurely. While the median duration of any BF was identical for the children ranked 1 and for the others, after 4-5 months, the proportion of children ranked >1 still being breastfed was higher (Figure 2). The gestation period had no significant impact on the duration of exclusive BF. Being born in a BFHI-accredited maternity unit did not influence the duration of either exclusive or any BF. The same can be said for the type of delivery and the sex of the child. Children born during the warmer months were breastfed longer than those born in autumn (Table 1).

The median duration for exclusive and any BF was longer for mothers who were foreign born, those who knew the WHO recommendation, those who had chosen to breastfeed before getting pregnant, those who had taken parental leave, and those who were unemployed. The mother's age only seemed to have an impact on any BF. The median duration increased with the mother's level of education but, overall, there was no significant difference between the curves (Table 2).

The father's positive attitude resulted in a longer median duration for both types of BF. Only the median duration of exclusive BF was longer when the level of household income was higher. The duration of BF was shorter (3 months) when the father had studied as far as upper secondary level. The median duration increased with the father's level of education but, overall, there was no significant difference between the curves (Table 3).

Table 4 shows the factors associated with the risk of stopping any BF and exclusive BF using Cox proportional hazards regression. Mothers who knew the WHO recommendation, women who prenatally intended to breastfeed, mothers who returned to work 3 months after the birth, and mothers of foreign origin were less likely to stop any BF and exclusive BF than other mothers. Women who perceived their partners to prefer BF and mothers who were 30 years old and over were less likely to discontinue any BF. The introduction of breast-milk substitutes before discharge from the maternity unit was significantly associated with a shorter “any BF” duration. When parental income was higher than €2000 or when mothers gave birth between May and September, children were significantly less likely to stop being exclusively breastfed than others.

## 4. Discussion

Around ten years ago, Cattaneo noted that Belgium was one of four countries in Europe that faced the biggest challenges, particularly as regards breastfeeding data collection [3]. Indeed, we can see that in Belgium (i) the only BF data available is collected by the BFHI program and by the Maternal and Child Health program (MCH), which only concern BF in the maternity unit; (ii) the BF definitions used are still fairly unclear; (iii) there is no representative data of BF duration; and (iv) it is impossible to cross-reference existing data with parents' sociodemographic characteristics.

Given these four issues, in 2012, a module comprising 16 retrospective questions on BF was added to the regional vaccination coverage survey. This data has several advantages. Firstly, it is a representative sample of the infant population of Wallonia. Secondly, the questions make it possible to identify the factors associated with weaning and to obtain their adjusted effects. They also make it possible to obtain the prevalence at specific moments in order to correlate with the WHO [15] and OECD [5] indicators.

### 4.1. Main Results

Exclusive BF at birth in the vaccination coverage survey fell from 73.1% at birth to 66.4% at discharge from the maternity unit (*P* < 0.01). This finding demonstrates the relevance of being able to measure prevalence at these two different moments and to review the capacity of health-care professionals to encourage and support new mothers to start and continue breastfeeding during the first few days following the birth.

In 2012, the data from the survey showed a prevalence of any BF of 81.7% at birth, 76.4% at discharge from the maternity unit, 54.1% at 3 months, 29.1% at 6 months, and 10.8% at 12 months. In a previous survey in 2009, four dichotomous questions were asked concerning the prevalence of any BF at these four moments. The values obtained were 75.7% (at birth), 48.3%, 26.0%, and 10.0%, respectively [16]. Prevalence in the maternity unit (at birth) and at 3 months, therefore, significantly increased compared with the 2009 vaccination coverage survey. When comparing with the data given by Cattaneo et al. for the whole of Belgium, which for any BF was 30–37% at 3-4 months, 10% at 6 months and 4% at 12 months [4], there has been a marked increase in any BF at 6 and 12 months since the 2000s.

### 4.2. Significant Role of the Father's Attitude and Knowledge of WHO Targets

Longer BF duration is associated with better maternal infant feeding knowledge [17, 18]. In Wallonia, mothers who knew the WHO BF recommendation breastfed significantly longer than those who did not. Many studies among first-time mothers have shown that maternal awareness in the antenatal period of the recommendation to breastfeed exclusively to age 6 months was significantly and positively associated with initiation and duration of any BF [17, 19]. Only 26% of mothers knew the WHO recommendation in our study. It is surprising to note that parental knowledge concerning WHO guidelines was no greater when the mother gave birth in a BFHI-accredited maternity unit. A large number of studies have shown the influence of the father's attitude toward breastfeeding on BF initiation and duration [9–12].

### 4.3. Relationship between Prenatal Intention to Breastfeed and Duration of Breastfeeding

The majority (65.2%) of the mothers intended to breastfeed before pregnancy. The median duration of exclusive BF was 3.5 months (3.2–3.8) for women intending to breastfeed before pregnancy and 2.0 months (1.5–2.5) for other women. For any BF the median duration was 4.5 months (3.9–5.0) and 3.0 months (2.5–4.5), respectively. Many studies have shown that women prenatally intending to breastfeed are more likely to initiate and continue BF [10, 20–23]. In Wallonia, as in other studies, the decision period is associated in the Cox model with the duration of any BF and exclusive BF.

### 4.4. The Negative Influence of Breast-Milk Substitutes at Discharge from the Maternity Unit

This study points to a strong negative association between the introduction of breast-milk substitutes at discharge from the maternity unit and the total length of any breastfeeding. A difference of 1.5 months can be seen between the two groups. In 1988, a survey of 5,367 consecutive hospital births in one of the provinces of Belgium (Hainaut) was carried out and showed the same result [24]. Since then, other authors have shown the negative influence of introducing early supplementary breast-milk substitutes in the maternity unit on BF duration [11, 25] and also on the difficulty of meeting the mother's own BF goals [20]. In this respect, only one-third of the mothers in our study (35.1%) were satisfied with their “any BF” duration.

A study carried out in Flanders in 2006 [26], among various categories of health-care professionals, showed that gynecologists, general practitioners, and pediatricians were not sufficiently aware of the negative impact of supplementation in the maternity unit on early weaning. As such, training should be introduced for all health-care professionals in contact with new and expectant mothers so that they are aware of the major impact of supplementation and also of the significant role of an early decision made jointly with the child's father. Indeed, it is important to note that health-care professionals appreciate the influence that positive and active partner support can have on promoting maternal confidence in breastfeeding [27]. Before the mother gives birth, parents should know (i) the WHO guidelines and the reasons for these recommendations; (ii) the negative impact of introducing breast-milk substitutes in the maternity unit and also before 6 months of age on breast-milk production; (iii) all the benefits of BF for both mother and child.

### 4.5. Important Role of the Sociodemographic Characteristics of the Parents

In our study, 21% of the women had returned to work at 3 months or earlier and over 45% of the women had extended their maternity leave with parental leave. Women who returned to work at 3 months or earlier were breastfeeding shorter (exclusively or any BF) than women who returned to work after 3 months. This negative association between early return to work and BF duration has been reported in many studies in industrialized countries [6, 11, 12, 22]. Belgian maternity leave is 15 weeks and is one of the shortest in Europe [5]. This maternity leave is not full pay.

As in a number of other studies, the mother's nationality of origin has a major influence, in Wallonia, on any BF and exclusive BF [6, 9, 12]. Our research, as many studies, suggests that women from lower socioeconomic groups wean earlier, mainly for exclusive BF [6, 8, 28]. Another observation frequently made is that younger mother's breastfeed for less time than older mothers [12, 17, 28, 29]. A number of researchers have also shown that educated women breastfeed for significantly longer than less-educated women [6, 28, 30]. Despite this, this variable has remained constant in BF studies; the mother's level of education is not associated with BF according to Cox modelling in Wallonia.

Concerning socioeconomic characteristics, the French “Eden” study conducted by Bonet et al. [12] reported results that were fairly similar to our own, even though the analyses were conducted by logistic regression of the prevalence of any and exclusive BF at 4 months. The author reported that the mother's nationality of origin, the duration of their maternity leave, and income are associated with exclusive BF at 4 months, while parity and the mother's age are not associated after adjustment for the other terms of the model. Age, nationality, and maternity-leave duration are associated with any BF as in our study. In Bonet's study, the mother's level of education, and income are associated with any BF, while in our study, these two factors are not associated after adjustment for the other predictors.

### 4.6. Minimal Impact of Birth-Related Characteristics on BF Duration

We showed a shorter “any BF” duration in children born prematurely. This difference is not demonstrated for exclusive BF. This has been confirmed elsewhere [18]. As certain authors have shown [11], the method of delivery has no impact on the duration of BF, unlike in other studies which show early weaning in the event of cesarean [9, 10].

Various authors show more frequent breastfeeding in the maternity unit in primiparous mothers and BF of longer duration in multiparous mothers [11, 20, 30–32]. The difference in the survival curves in Graph 2 demonstrates this. While the survival curves overlap initially, the curves diverge when 50% of the children have been weaned. Consequently, the curves show earlier weaning in children ranked 1. Due to this trend reversal over time, comparison with studies based on BF prevalence at fixed moments (e.g., at 1 or 3 months) can give logistic regression associations that are different from ours [12, 28]. Ingram et al. wrote that significantly more breast milk was produced at 1 week and at 4 weeks for the second lactation and the net increase was greatest for those with the lowest milk output on the first occasion. Mothers spent less time feeding their second baby [33].

Nearly one-quarter (23.5%) of the children in our sample were born in a BFHI-accredited maternity unit. One could have expected, as other studies have shown, median BF durations to be longer for children born in BFHI-accredited maternity units [13]. We think that a large majority of maternity units are aware of the clear benefits of BF. Maternity units can have a BF support policy without being a BFHI-accredited hospital and can deliver messages that are effective for both BF initiation and BF duration. We have also shown that there is no difference in knowledge between the two types of maternity units as regards duration targets.

### 4.7. Limitations of the Study

At the time of the investigation all the children were aged between 18 and 24 months. Thus, there might have been some recall bias. However, previous studies indicate that the recall of initiation and duration of breastfeeding are more accurate than recall of when foods were introduced [34, 35]. In 2002, in the USA, the prevalence of initiation and duration of breastfeeding was also analyzed using the coverage survey with the same potential bias [31].

We do not know anything about the profile of parents refusing to participate in the study. We do not know if such refusal is linked with a negative attitude toward BF. However, we believe that this bias is limited because the parents did not know the exact topic of the survey before agreeing or refusing to participate. They were informed that it was a survey on infancy. Children residing illegally in Wallonia were not included in the survey.

## 5. Conclusions 

Programs to promote and support BF should concentrate on better training for health-care professionals involved in prenatal care. Professional advice that focuses on prenatal maternal knowledge may promote adherence to WHO BF guidelines, and particular emphasis should be placed on the benefits of exclusive BF. Women should be discouraged during pregnancy from introducing breast-milk substitutes while in the maternity ward. In Wallonia, the rate and duration of BF are still the lowest in Europe, just as maternity leave is the shortest in Europe. Developing a breastfeeding culture that also involves increasing the duration of maternity leave would be a consideration.

## Figures and Tables

**Table 1:** Characteristics of the children and median duration of any and exclusive BF (in months) with 95% confidence interval (CI), *P* values.

	% (*n*)	Any breastfeeding			Exclusive breastfeeding		
		Median duration	95% CI	*P* value	Median duration	95% CI	*P* value
BF at discharge maternity unit							
Exclusive	87.0 (348)	4.5	4.0–5.0	<0.001			
Partial	13.0 (52)	3.0	2.0–4.0				
First-born infant							
Yes	42.9 (225)	4.0	3.5–4.5	0.01	3.0	2.5–3.4	0.08
No	57.1 (299)	4.0	3.3–4.7		3.0	2.7–3.3	
Full term							
Yes	92.2 (484)	4.0	3.5–4.5	0.04	3.0	2.8–3.2	0.8
No	7.8 (41)	3.5	2.0–5.0		2.0	0.8–3.2	
Delivery in BFHI							
Yes	23.5 (118)	3.0	2.3–3.7	0.3	3.0	2.5–3.5	0.2
No	76.5 (385)	4.0	3.4–4.6		3.0	2.7–3.3	
Mode of delivery							
Vaginal	78.2 (410)	4.0	3.5–4.5	1.0	3.0	2.7–3.2	0.6
Cesarean section	21.8 (114)	4.5	3.6–5.4		3.0	2.0–4.0	
Infant gender							
Male	51.8 (271)	4.0	3.2–4.8	0.5	3.0	2.7–3.3	0.9
Female	48.2 (252)	4.0	3.3–4.6		3.0	2.5–3.5	
Month of birth							
May–September	66.3 (349)	4.5	4.0–5.0	0.03	3.0	2.7–3.3	0.01
October-November	33.7 (177)	3.0	2.3–3.7		2.0	1.5–2.5	
MCH attendance							
Yes	72.6 (382)	4.0	3.5–4.5	1.0	3.0	2.8–3.2	0.6
No	27.4 (144)	4.5	3.6–5.4		3.0	2.5–3.5	

**Table 2:** Maternal characteristics and median duration of any and exclusive BF (in months) with 95% confidence interval (CI), *P* values.

	% (*n*)	Any breastfeeding			Exclusive breastfeeding		
		Median duration	95% CI	*P* value	Median duration	95% CI	*P* value
Native nationality							
Belgian	71.8 (377)	3.5	3.1–3.8	<0.001	3.0	2.7–3.3	<0.001
Foreign	28.2 (148)	6.0	5.4–6.6		4.0	3.3–4.7	
Awareness WHO recommendation							
Yes	27.5 (132)	6.0	5.2–6.8	<0.001	4.0	3.5–4.5	<0.001
No	72.5 (348)	3.5	3.1–3.9		3.0	2.6–3.4	
Prenatal maternal intention							
Yes	64.5 (300)	4.5	3.9–5.1	0.006	3.0	2.7–3.3	0.03
No	35.5 (165)	3.0	2.4–3.6		2.0	1.5–2.5	
Age (years)							
16–29	39.9 (205)	3.5	3.0–4.0	0.05	3.0	2.4–3.6	0.3
30–47	60.1 (309)	4.5	3.8–5.1		3.0	2.6–3.4	
Education level							
≤first 3 years of secondary school	34.6 (178)	3.0	2.5–3.5	0.09*	3.0	2.5–3.5	0.2*
Last 3 years of secondary school	23.3 (120)	4.0	2.3–5.7		3.0	2.3–3.7	
Higher education	42.1 (217)	4.5	4.0–5.0		3.0	2.6–3.3	
Age of infant when mother returned to work							
≤3 months	21.0 (109)	3.0	2.5–3.5	<0.001	2.0	1.5–2.5	<0.001
4 months and more	45.6 (236)	4.5	4.0–5.0		4.0	4.0–4.3	
Unemployed	33.4 (173)	4.0	2.7–5.3		3.0	2.0–4.0	

*Breslow test ≤ 0.05.

**Table 3:** Paternal characteristics and median duration of any and exclusive BF (in months) with 95% confidence interval (CI), *P* values.

	% (*n*)	Any breastfeeding			Exclusive breastfeeding		
		Median duration	95% CI	*P* value	Median duration	95% CI	*P* value
Education level							
≤first 3 years secondary school	40.0 (193)	4.0	3.4–4.6	0.5*	3.0	2.7–3.3	0.7
Last 3 years of secondary school	22.8 (110)	3.0	2.3–3.7		2.0	0.7–3.3	
Higher education	37.2 (180)	5.0	4.4–5.6		3.0	2.6–3.4	
Partner's attitude							
Positive	75.6 (377)	4.0	3.5–4.5	0.006	3.0	2.7–3.3	0.02
Indifferent/negative	24.4 (122)	2.0	1.3–2.7		2.0	1.0–2.9	
Household income (Euros)							
≤2000	37.7 (177)	4.0	3.3–4.6	0.9	2.0	1.5–2.5	0.03**
>2000	62.3 (293)	4.0	3.5–4.5		3.0	2.5–3.4	

*Breslow test ≤ 0.05; **Breslow test ≤ 0.01.

**Table 4:** Results of Cox's proportional hazards models (HRs, 95% CI) for discontinuing any and exclusive BF, *P* values.

Variables in the model	Any breastfeeding (*n* = 303)			Exclusive BF (*n* = 286)		
	aHR	95% CI	*P* value	aHR	95% CI	*P* value
Mother's native nationality						
Belgian	1		0.006	1		0.002
Foreign	0.7	0.5–0.9		0.6	0.5–0.8	
Prenatal maternal intention						
Yes	0.8	0.6–1.0	0.04	0.7	0.6–0.9	0.02
No	1			1		
Awareness of WHO recommendation						
Yes	0.5	0.4–0.7	<0.001	0.7	0.6–1.0	0.03
No	1			1		
Age of infant when mother returned to work						
≤3 months	1			1		
>3 months	0.4	0.3–0.5	<0.001	0.6	0.4–0.8	0.002
Unemployed	0.5	0.4–0.7	<0.001	0.7	0.5–1.1	0.09
BF at discharge from maternity unit						
Exclusive	0.5	0.3–0.7	<0.001			
Partial	1					
Mother's age						
16–29	1			1		
30–47	0.7	0.5–0.9	0.02	0.9	0.7–1.2	0.7
Partner's attitude						
Positive	0.7	0.5–1.0	0.02	0.8	0.6–1.2	0.3
Indifferent/negative	1			1		
Household income (Euros)						
≤2000	1			1		
>2000	1.0	0.8–1.4	0.7	0.8	0.6–1.0	0.1
Month of birth						
May–September	0.8	0.6–1.0	0.06	0.8	0.6–1.0	0.08
October-November	1			1		
First-born infant						
Yes	1			1		
No	0.9	0.7–1.1	0.2	0.9	0.7–1.1	0.3
